# Presence of transmural posterolateral scar by LGE MRI is associated with non-response to CRT

**DOI:** 10.1186/1532-429X-13-S1-P256

**Published:** 2011-02-02

**Authors:** Leslie Chan, Jonathan Suever, Brandon Fornwalt, Stephanie Clement-Guinaudeau, Antonello D'Andrea, Luca Del Viscovo, Frits Prinzen, Frank Bracke, Angel Leon, David Delurgio, Michael Lloyd, John Oshinski

**Affiliations:** 1 Emory University School of Medicine, Atlanta, GA, USA; 2 Georgia Institute of Technology / Emory University, Atlanta, GA, USA; 3 Second University of Naples, Naples, Italy; 4 Maastricht University, Maastricht, Netherlands; 5 Catharina Hospital Eindhoven, Eindhoven, Netherlands

## Objective

Evaluate the effect of myocardial scar burden, scar location, and scar transmurality on response to cardiac resynchronization therapy (CRT)

## Background

Several recent single center studies have used late Gadolinium enhancement (LGE) MRI to predict which patients would likely not respond to cardiac resynchronization therapy (CRT). Results from these studies have varied on the importance of scar location, transmurality, and burden on response to CRT. We analyzed cardiac MRI in patients undergoing CRT from 4 centers (two in Europe and two in the US), and hypothesized that the presence of transmural scar in the posterolateral wall (the most frequent site of left ventricular lead placement in CRT) would preclude positive response to CRT.

## Methods

Cardiac MRI was performed according to standard methods and included at a minimum, cine imaging and LGE imaging using a Gadolinium-based contrast agent. All patients met current criteria for CRT (LVEF<35%, QRS>120 msec). Sixty (60) patients from 4 centers were analyzed, and any patient with infarct enhancement on LGE by visual inspection was processed into an LGE bullseye map following AHA guidelines. Total scar burden (as % of LV mass) as well as the presence of transmural scar in the posterolateral or septal segments were assessed. 48% of patients (29/60) patients had LGE and 59% of those (17/29) had transmural scar in at least one AHA segment. Positive response was assessed at 6 months both by clinical (increase of 20% in 6 minute hall walk distance) and echocardiographic (reduction of LVESV by 15%) parameters.

## Results

None of the patients with transmural *posterolateral* scar responded by clinical criteria, and only one patient with *posterolateral* scar responded by echocardiographic criteria (negative predictive value, NPV = 90%). The negative predictive value for the presence of transmural *septal* scar or total scar burden >15% were lower (NPV = 64-67%).

## Conclusions

The presence of transmural posterolateral scar by LGE is predictive of non-response to CRT.

